# Conflict within the Genome: Evolving Defenses to Suppress the Male Killers

**DOI:** 10.1371/journal.pbio.0040308

**Published:** 2006-08-22

**Authors:** Liza Gross

In the game of survival, anything goes even the selective extermination of males. Male killing is the preferred strategy for a diverse group of bacteria that infect insects and other arthropods. Aside from its tabloid appeal, male killing offers biologists a platform for investigating genetic conflict evolutionary battles between competing elements within the same genome. Male-killing bacteria are passed from mother to offspring, but only males die from infection, suggesting that males harbor genetic elements that allow them to succumb to infection. In keeping with evolutionary theory, these selfish genetic elements, which spread at the expense of the organism, should engender counteracting elements that promote male survival. Yet scant evidence has linked the evolution of host suppressors to selfish elements that mediate male killing.

But now, Emily Hornett, Gregory Hurst, and colleagues report the first case of total suppression of male killing in a butterfly, *Hypolimnas bolina*, infected with the *w*Bol1 strain of the male-killing bacterium *Wolbachia*. They attribute survival to genetic elements expressed in the male embryo, an effect called zygotic suppression. Because this mechanism of suppression can inactivate male killers which lie dormant until presented with a novel, vulnerable host it's possible that insects that don't succumb to male killing today may have in fact evolved the means to counteract lethal infection.

*H. bolina* is found throughout the Indo-Pacific. Because *w*Bol1 infection kills males in Polynesia but not in Southeast Asia, breeding individuals from each region could reveal genetic elements in the different populations that favor life over death. And because infected females transmit infection directly to offspring, breeding could also introduce *w*Bol1 genes (and infection) onto the butterfly genetic background (a technique called introgression).

The breeding experiments tested two questions: would male-killing *w*Bol1 taken from Moorea in Polynesia lose that ability against Southeast Asian males with a Thai or Philippine genetic background, and would benign *w*Bol1 from Thailand or the Philippines turn lethal against males with a Moorean genetic background?

To find out, Hornett et al. mated infected Moorean females with Thai and Philippine males, and mated infected Thai and Philippine females with Moorean males. As a control, *w*Bol1-infected females from both regions were also crossed with males from their native populations. Crossing the Moorean and Southeast Asian populations suppressed the male-killing effects of *w*Bol1 from Moorea in just a single generation in stark contrast to the control crosses (Moorean females mated with Moorean males), which yielded no males at all.

But when Moorean *w*Bol1 infection was reintroduced to its native host background by backcrossing first-generation hybrid Moorean/Southeast Asian females with wild Moorean males it became male-lethal again. Egg hatch rates decreased dramatically and just a fraction of males survived. In contrast, continued introduction of Moorean *w*Bol1 infection onto the Southeast Asian male genetic background produced high hatch rates and a normal sex ratio.

**Figure:**
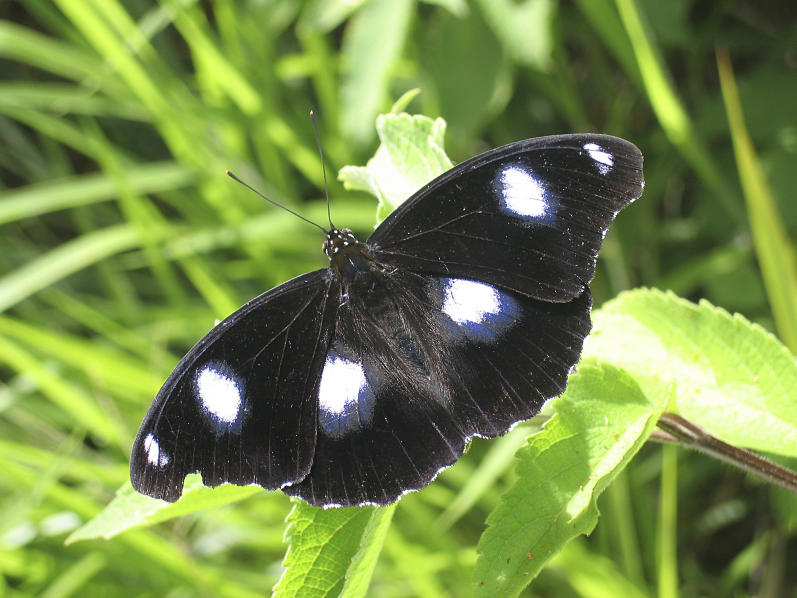
A male nymphalid tropical butterfly *Hypolimnas bolina*, otherwise known as the Common Eggfly. (Photo: Sylvain Charlat)

Infected Thai and Philippine females were serially mated with Moorean males, thus progressively increasing the proportion of Moorean genetic material. By the second generation, some male killing occurred, and by the third generation, males were killed in five out of 13 crosses. By the fifth generation, no males survived.

From these results, the researchers concluded that suppression occurs in the embryo, because male offspring of Moorean females crossed with the Southeast Asian males survived even though the mother's genetic profile allows killing. The fact that first-generation hybrids survived at nearly the same ratio as seen in wild Southeast Asian males, they explain, suggests the effect is dominant (requires just one copy of the gene) and is at high frequency in the population. A dominant effect also explains why male killing didn't occur in first-generation crosses between Southeast Asian females and Moorean males the suppressor elements had not been segregated out of the population yet.

Through simulations, the researchers show that the suppressor could spread through the population in just 100 generations, suggesting that male killing could disappear relatively quickly after a suppressor mutation occurs. Thus, genetic conflict between killing abettors and suppressors may be far more widespread than once thought, but has simply eluded detection. Given the diversity of species afflicted by male-killing bacteria, researchers will have plenty of options for testing this possibility.

